# Digital health interventions for improving nutritional behaviors in older adults: a scoping review

**DOI:** 10.3389/fnut.2026.1743189

**Published:** 2026-05-04

**Authors:** Alessandra Buja, Giulia Grotto

**Affiliations:** 1 Department of Cardiac, Thoracic and Vascular Sciences and Public Health, University of Padua, Padua, Italy; 2 Department of Pharmaceutical and Pharmacological Sciences, University of Padua, Padua, Italy

**Keywords:** digital technology, healthy diet, nutrition, nutritional interventions, older adults

## Abstract

**Background:**

In the context of an aging population, nutrition plays a fundamental role. Eating habits can change in old age due to physical disorders, social isolation, and decreased appetite, leading to a decline in nutritional status. The use of digital technologies has become widespread in recent years, even among older adults, and with it, digital nutrition interventions. The aim of this scoping review was to identify key characteristics of digital nutrition interventions for older adults, appraise currently available evidence of their effectiveness, and investigate users’ perceptions.

**Methods:**

PubMed, PsycINFO, CINAHL, and Scopus databases were searched for relevant studies in April 2025. Retrieved studies were evaluated for compliance with eligibility criteria, which involved selecting studies of nutritional interventions delivered through digital technologies and aimed at community-dwelling older adults. A descriptive analysis was performed to report the characteristics of the included studies and their outcomes.

**Results:**

Thirteen studies met the eligibility criteria and were included in the systematic review. Among the analyzed outcomes, digital nutrition interventions appeared effective in increasing dietary fiber intake, while no effect emerged on quality of life, appetite, or calorie intake. Mixed results were found regarding improvements in nutritional status, self-perceived health, adherence to the Mediterranean diet or other dietary guidelines, and protein intake. Dropout rates were generally high and tended to increase toward the end of the interventions. Qualitative analyses revealed some negative aspects such as the perceived complexity of using the apps, low acceptability, restrictions, and heavy burden brought into daily life by the interventions, and frustration derived from technical issues. Conversely, participants appreciated increased knowledge, greater awareness of nutrition, and motivational and inspirational content.

**Conclusion:**

Aside from dietary fiber intake, digital nutrition interventions did not prove effective in increasing nutritional behaviors in older adults.

## Introduction

The average age of the global population is increasing, and the number of people aged 60 and older is projected to grow from 1.1 billion in 2023 to 1.4 billion by 2030 (1). This demographic shift has significant implications for public health, as the adoption of lifelong health promotion and disease prevention strategies can significantly reduce the risk of developing non-communicable and chronic diseases, including heart disease, stroke, and cancer, as well as functional impairment, enabling older people to maintain good health, remain independent, and participate actively in family and community life (1–3).

Retirement and leaving the workplace can provide new challenges among older people and influence the development of NCDs: poor nutrition, lack of physical activity, social isolation, and financial stress directly affect older people and strongly promote NCDs (3). Eating behavior might change in older age; for instance, chewing and swallowing difficulties, weakened taste ability, and declining olfactory function might have an impact on older people’s appetite, food choice, and intake, leading to a lower micro- and macronutrient intake from food and to a declining nutritional status (4). For these reasons, and also due to decreased physical activity, older adults are more likely to lose lean body mass (5). Therefore, effective and advanced dietary interventions that ensure adequate nutrient intake in older adults, especially those affected by age-related diseases and who are isolated, are in high demand.

With the widespread diffusion of digital technology over the past few decades, the possibility has emerged to promote nutritional education by bringing information directly to users through information and communications technologies (6, 7). The multitude of health and diet-related apps offered on Google Play and the Apple App Store highlights the public’s increasing commitment to a healthy lifestyle (8).

Previous research showed that digital interventions can promote various health behaviors, including diet and physical activity, in the general population (6, 9). Among the prospective advantages of providing behavioral interventions through digital platforms are their capacity for widespread dissemination, the enhanced ease of access for diverse populations, their inherent ability to be expanded considerably, and the notable economic efficiency they offer (10, 11). Also, the use of communication technologies expands the possibilities for delivering alerts and messages to participants to support their adherence to interventions (10, 12). Despite their potential, interventions utilizing online platforms and smartphones often face challenges in fostering and preserving participant engagement, given that substantial attrition is a prominent issue in the field (13). In addition, the effectiveness of digital interventions for improving nutrition is still little studied among the older population. While in the early 2000’s the internet was used mostly by people aged 18–49, the proportion of over-65’s who surf the net has almost reached that of younger age groups, exceeding 80% (14). This has opened the possibility of developing digital health promotion interventions specifically aimed at the older population.

Most of the previous scientific research on this topic has focused primarily on analyzing the effectiveness of digital nutritional interventions for the general population but not on older adults (6, 9). The reviews that did specifically include studies on older adults had specific focuses, such as assessing the effectiveness of interventions aimed at increasing fruit and vegetable intake (15), improving malnutrition through telemedicine programs (16), or the effects of digital nutrition interventions in individuals with prediabetes (17). Consequently, there is a lack of reviews aimed at investigating digital nutrition interventions conducted on community-dwelling healthy older adults.

The objectives of this systematic review were (a) to identify the main characteristics of digital nutrition interventions for healthy, community-dwelling older adults, (b) to appraise the available evidence of their effectiveness in improving the nutritional status of the participants compared to control groups or baseline assessments, and (c) to evaluate users’ perceptions of these digital nutrition programs.

## Methods

This scoping review was conducted in accordance with the Preferred Reporting Items for Systematic Reviews and Meta-Analysis (PRISMA) guidelines (18). The review protocol was defined in advance and registered with the International Prospective Register of Systematic Reviews (PROSPERO – CRD420251080903).

### Search strategy

Four scientific databases (PubMed, PsycINFO, CINAHL, and Scopus) were searched on April 4th, 2025, for relevant primary studies. The search string was developed by combining keywords for the three main research axes (“older adults,” “digital technology,” and “nutrition”) and their respective synonyms using Boolean operators. For the PubMed database, a combination of Medical Subject Headings (MeSH) terms and text words was used. The search strings are detailed in Supplementary Appendix A. The retrieved studies were independently reviewed for eligibility by two authors (GG and AB) in a two-step process: an initial screening based on titles and abstracts, followed by full-text screening. Disagreements between the two reviewers were resolved by discussion until a consensus was reached.

### Inclusion and exclusion criteria

The review questions were predefined in advance using the PICO (Population, Intervention, Comparison, Outcome) framework (19). The population group was identified as community-dwelling older adults; the intervention consisted of automated nutritional programs delivered via digital technology (mHealth or eHealth); the comparison was control groups of older adults not receiving the nutritional intervention in controlled studies and the baseline assessments from before-and-after studies; outcomes of interest were changes in nutritional status, nutritional behaviors, and nutritional knowledge of the participants, and users’ perceptions of the digital technology interventions. Studies were included in the systematic review if they met the following criteria: (a) performed an evaluation of the effectiveness and/or users’ perceptions of digital nutrition interventions (eHealth or mHealth); (b) the study sample included community-dwelling older adults with a mean age of 60 years or more; (c) digital interventions were conducted using automated tools (i.e., apps or online platforms) entirely or for most of the intervention. Blended interventions that involved brief contact with staff (e.g., in the initial or final phases) were included only if the majority of the intervention was automated. Nutritional interventions led exclusively by health professionals via telemedicine, without any automated components, were excluded; (d) were published in the last 10 years (2015 onwards); (e) were written in English; (f) were published in a peer-reviewed scientific journal.

Studies were not considered for inclusion if they were: (a) conducted on participants affected by a specific disease or medical condition (i.e. interventions for older obese adults aimed at weight loss); (b) conducted on older adults admitted to hospitals, nursing homes, or residential facilities; (c) designed to target the general population, and not specifically older adults; (d) non-interventional studies (i.e., guidelines, reviews, letters, and editorials).

### Data extraction and data analysis

A data extraction form based on the research question was developed in Microsoft Excel. The following data were extracted from the included studies: (a) main author, (b) year, (c) sample size, (d) country, (e) mean age, (f) objectives, (g) delivery modes (automated or blended), (h) description of the intervention, (i) duration, (j) study design, (k) outcomes: efficacy, (l) outcomes: users’ perceptions. A descriptive analysis was performed to report the characteristics of the included studies (Table 1) and their outcomes (Table 2).

**TABLE 1:** Characteristics of the included studies.

References	Country	Sample size (N), sex	Age	Delivery modes of the intervention (automated or blended)	Duration	Study design
Aakre et al. (28)	Norway	16, 7 women	Age range 67–74: 6 participants; above the age of 75: 10 participants	Blended	2 days in person + 9 months follow up	Qualitative design: three focus groups using a semi-structured interview guide
Bohn et al. (32)	Portugal, Netherlands	107 in Netherlands field trial (48% women) 53 in Portugal (73% women)	Portugal field trial: mean 69 Netherlands field trial: mean 72	Automated	14 months in the Portugal field trial, 3 months in Netherlands field trial	Mixed method: non-controlled pre-post-test study, questionnaires and qualitative feedback (focus groups and workshops)
Chiu et al. (24)	Taiwan	21, 52.4%women	Mean 65 (SD 8.33)	Blended	6 weeks	Mixed method: non-controlled pre-post-test study and qualitative investigation
Doets et al. (22)	Netherlands	59, 37 women	Mean 67.7 (SD 4.8)	Blended	9 weeks	Quantitative: randomized controlled trial
Dorhout et al. (25)	Netherlands	19, 14 women	Mean 69 (SD 7)	Blended	24 weeks	Mixed method: non-controlled pre-post-test study and qualitative (semi structured interviews)
Farsjø Aure and Moen (29)	Norway	4, all women	Age range: 69–76	Blended	4 weeks	Qualitative design: a focus group guided interview
Farsjø Aure et al. (30)	Norway	18, 12 women	Mean 81, age range: 68–95	Blended	8 weeks	Qualitative design: semi-structured interviews
Farsjø Aure et al. (31)	Norway	25, 18 women	Mean 79, age range: 68–95,	Blended	8 weeks	Qualitative design: semi-structured interviews and descriptive analysis of log data
Gomes et al. (33)	Portugal	31, 21 women	Mean 71.9 (SD 6.6)	Automated	12 weeks + 3 months follow up	Quantitative: non-controlled pre-post-test study
Recio-Rodriguez et al. (34)	Spain	160, 61,3% women	Mean 70.8 (SD 4)	Blended	3 months	Quantitative: randomized controlled trial
Van Asbroeck et al. (26)	Netherlands	477, 70.9% women	Mean 63 (SD 12)	Automated	7 weeks	Quantitative: non-controlled pre-post-test study
van Doorn-van Atten et al. (27)	Netherlands	214, 146 women	Mean 80	Blended	6 months	Quantitative: controlled pre-post-test study
van Doorn-van Atten et al. (23)	Netherlands	20, 15 women	Mean 80.6 (SD 8.4)	Blended	3 months	Mixed method: non-controlled pre-post-test study and qualitative (interviews and evaluation meetings)

**TABLE 2:** Objectives and findings of the included studies.

References	Objectives	Intervention’s description	Outcomes: efficacy	Outcomes: users’ perceptions
Aakre et al. (28)	(1) To conduct a health-promoting and disease-preventing nutrition education intervention with a theoretical, practical, and digital aspect aimed at healthy community-dwelling older adults; and (2) To evaluate and describe its feasibility through the participants’ experience with the intervention.	Two consecutive days of in-person meetings covered nutrition/health content, a cooking class, and an app introduction with indefinite access. Specifically created for seniors, the app offered features like daily meal plans with appealing food images. It allowed users to log food and drink intake, adjusting portion sizes. Daily energy, protein, and fluid consumption were displayed visually on a human figure and via graphs comparing it to recommended levels. Positive reinforcement, a smiling figure and cheering sound occurred upon meeting dietary targets.	–	Though somewhat relevant and inspiring, most participants neglected the app, deleting it soon after the intervention. Its complex, time-intensive nature led to a consensus that it was better for future generations. Some of the participants said that they used the app occasionally to check their nutritional intake.
Bohn et al. (32)	To develop a mobile nutrition solution, i.e., a dietary meal-recommender app for personalized meal planning, useful for older adults. The general aim of the field trials was to study the effect of using the LIFANA solution in healthy seniors longitudinally over time, regarding acceptance of the solution and to monitor their weight as primary outcomes.	A meal recommender app aimed at providing older adults with regular balanced meal suggestions (meal planning), personalized for their age, gender, physical activity level, personal food restrictions and preferences, and considering overall calorie and protein targets (protein goal was set at 0.8 g/kg body weight).	23 and 34 older adults finalized the trials in Portugal and Netherlands, respectively. No significant changes in height, weight, body fat, waist-hip circumferences, BMI, and blood pressure, were observed.	Portugal. 47% of users reported that they might use the solution if it became available. 36% dropout rate. Acceptability: among 15 participants, six maintained a neutral stance in their answers over time, seven became more negative, and two showed increased positivity (higher agreement). Meal creation rose from 20 to 30 to over 60 by the trial’s end. Netherlands. Usage: 86% were online daily. The rest went online a few times per week (10%), weekly (3%) or less often (1%). Acceptability: of 23 individuals, one maintained a neutral stance, 20 developed more negative responses, and two developed more positive agreement over time. A total of 9% of users reported that they might use the solution if it became available. A total of 68% drop-out rate. Early in the trial, 20–40 meals were created, but this decreased to under 10 daily later.
Chiu et al. (24)	To determine whether (1) nutrition education combined with mobile technology- supported teaching improves knowledge of and self-efficacy for a healthy diet, and (2) if adults who reported reviewing the electronic course material or searching health information online showed significantly greater progress in knowledge of and self-efficacy for a healthy diet than did those who did not adopt the electronic support.	The intervention included two facets: (1) a dietitian’s three-session traditional nutrition lecture with paper course material; (2) a research assistant’s three-session touch-screen tablet-computer-supported instructions on how to use the Internet and how to search for health-knowledge applications. Each type of instruction was provided every other week for 90 min. All the participants were given both types of intervention. Also, participants were given a tablet computer with more than 50 downloaded nutrition-related films and nutritional apps on it.	Nutrition knowledge significantly improved (mean post-pre = 1.19, *p* = 0.001) and was positively correlated with their intensity of surfing the Internet (*r* = 0.46, *p* < 0.05) and with reviewing the electronic course material (*r* = 0.48, *p* < 0.05), but not with reviewing paper course material (*r* = 0.19, *p* = 0.09). Self-efficacy about a healthy diet showed marginal improvement (mean post-pre = 0.22, *p* = 0.07).	Qualitative results showed that participants reported feeling freshness, joyfulness, and great achievement because of the combined course material. The participants agreed that they were able to learn specialized nutritional knowledge in lectures given by professional dietitians, but when using a touch-screen device to learn about improving their own everyday diet, they felt that the knowledge they acquired was more important. Engagement: middle-aged participants reported higher frequency of reviewing electronic course materials than older participants.
Doets et al. (22)	To evaluate whether personalized as compared to generic lifestyle advice improves wellbeing of seniors in terms of self-perceived health (primary outcome) and objective biological health measures (secondary outcome).	Initially, participants received health feedback from thorough baseline measurements. The intervention group (*n* = 30) received the same food-based dietary guideline leaflet as the control group, plus personalized online advice. This advice included seven dietary points (protein, energy, saturated fat, omega-3, salt, vitamin D, non-alcoholic liquid) and two physical activity points (aerobic, resistance exercise). Each tailored recommendation featured a green, orange, or red smiling emoji, indicating whether behavioral adjustment for that area was low, moderate, or high. Participants had to create action plans detailing how they would use at least two pieces of advice, especially those marked with orange or red emojis, through a digital survey offering time, situation, and action choices.	Behavior change: at the study’s start, about half the participants needed to adjust their intake of calories, saturated fat, omega-3’s, and vitamin D. Intake of omega-3’s, saturated fat, and liquids improved. The dietary advice failed to effectively increase calorie and vitamin D intake. High initial intentions for protein consumption did not prevent its decline during the study (*n* = 14). No differences were found between the intervention and control group in perceived compliance with Dutch food based dietary guidelines and perceived healthiness of actual diet at the end of the study. Self-efficacy regarding protein intake tends to decrease over the course of the intervention period (−0.5 CI: −1.0, −0.1); other health measures (self-perceived health, SPF-12 physical health, SPF-12 mental health) did not change over time. The intervention group showed a reduction in waist circumference (−1.9 CI: −2.9, −0.8).	Some participants (*n* = 8) experienced difficulties with the digital food diary, especially as the application’s user-friendliness received a low score. In general, all participants evaluated the study as very positive and all but one indicated they would participate again. The intervention group indicated they increased their awareness, knowledge, and insight into individual health behaviors. Mean self-reported compliance with the formulated implementation intentions was 4.86 (seven-point scale, SD = 1.46).
Dorhout et al. (25)	To investigate the effects of a web-based lifestyle intervention on muscle strength, protein intake, and physical functioning in healthy older adults.	Participants performed resistance training at home twice a week for 24 weeks via web-based workout videos. In addition, older adults were advised on increasing protein intake via two web-based consultations by a dietitian in the first 12 weeks and via an e-learning course in the second 12 weeks. The nutrition module consisted of 12 submodules, with a new submodule released every week. Various themes related to proteins were discussed in the form of videos, short texts, assignments, quizzes, and recipes. Each participant accessed the module with a personal account and performed it at their own pace.	Total protein intake per day did not change.	The web-based nutrition module was rated 7.1 on average (scale 1–10); on average, 80% of the participants performed the first six modules, and 60% of the participants conducted the last six modules. The level of difficulty for the modules was considered suitable. However, some participants thought it should be more challenging. Participants appreciated the schedule of the nutrition module, with a new module being presented on the web every week. In addition, the different elements of the nutrition module (knowledge clips, text, quizzes, and recipes) were mentioned as assets. A feature that should be added to the nutrition module is the possibility to have a web-based conversation to gather experiences from other users.
Farsjø Aure and Moen (29)	To assess if the application was sufficiently adapted to elderly, inexperienced users of technology and if it had potential to contribute to encouragement and orientation about meals.	A tablet application about nutrition, presenting dishes, enabling registration of food choices and notifications about mealtime was developed specifically for older adults.	–	Meal suggestions presented as pictures inspired and influenced to some extent the participants’ meal choices. Two participants found that reporting food choices could increase awareness, while two saw little need for this function in the prototype. The participants used APPETITT daily and considered it easy to use. The pilot test revealed that notification of meals did not work as anticipated; only one of them had paid attention to the notifications.
Farsjø Aure et al. (30)	To investigate older adults’ experiences of using the Appetitus app with support from healthcare professionals.	Appetitus was developed as a tablet-based application to prevent and alleviate undernutrition among older adults: pictures of meals and beverages sought to present common, varied and easily available meals in appetite-friendly presentations; users could record their food and beverage consumption and adjust the portion size to better reflect consumption when calculating nutritional value; users who reached their energy and fluid goals for the day received feedback in the form of a full figure smiling and making a cheering sound. Older adults received home care, and local healthcare professionals introduced the app and gave support during the study.	–	Appetitus served as a source of inspiration and a reminder of available, relevant food options. Appetitus encouraged some participants to eat or drink more by the end of the day while others became more aware of selecting food options to ensure sufficient protein, energy, and fluids. However, some participants made no active effort to change their diet despite feedback from the app that suggested they did not eat or drink enough. Technical support from healthcare professionals facilitated participants’ use of the app and tablet. Some participants also received more specific nutritional follow-up that helped to make their experience of using the app more meaningful.
Farsjø Aure et al. (31)	A feasibility study aimed at exploring older adults’ use of a nutrition app called Appetitus and addressing their engagement in technology mediated self-monitoring of diet.	Appetitus was developed as a tablet-based application to prevent and alleviate undernutrition among older adults: pictures of meals and beverages sought to present common, varied and easily available meals in appetite-friendly presentations; users could record their food and beverage consumption and adjust the portion sizes to better reflect consumption when calculating nutritional value; users who reached their daily energy and fluid goals received feedback in the form of a full figure smiling and making a cheering sound. Older adults received home care, and local healthcare professionals introduced the app and gave support during the study.	–	Most participants used the Appetitus app for 8 weeks, though usage declined in the last weeks. While recording increased food awareness and was generally easy, some found it restrictive and a burden. The project boosted many users’ tech confidence and encouraged further technology adoption.
Gomes et al. (33)	To explore the feasibility and acceptability of a home-based eHealth intervention focused on improving dietary and physical activity through an interactive television (TV) app among older adults with food insecurity.	An interactive TV app delivers a home program for older adults, promoting healthy living. It educates on diet and exercise, shows low-cost healthy options, and motivates adopting these habits to cut disease risk. Weekly interactive TV reminders with healthy lifestyle tips were sent to boost participant motivation. The TV app provided brief questionnaires to gauge participants’ adherence and understanding throughout the intervention. Weekly questionnaires were answerable via TV remote buttons.	Food insecurity (Household Food Insecurity Scale): reduction of 40% (*P* = 0.001) Quality of life (European Quality of Life Questionnaire with five dimensions and three levels): no differences; Fatigue (Functional Assessment of Chronic Illness Therapy-Fatigue) decreased (mean -3.82, SD 8.27; *P* = 0.02) Physical function (Health Assessment Questionnaire) improved, mean -0.22, SD 0.38; *P* = 0.01. Adherence to the Mediterranean diet: no difference	Feasibility (self-reported use and interest in eHealth): 10 participants self-reported low use of the TV app. After the intervention, participants were significantly more interested in using eHealth to improve food insecurity (baseline median 1.0, IQR 3.0; 3-month median 5.0, IQR 5.0; *P* = 0.01) and for other purposes (baseline median 1.0, IQR 2.0; 3-month median 6.0, IQR 2.0; *P* = 0.03). Acceptability (affective attitude, burden, ethicality, perceived effectiveness, and self-efficacy): high levels of acceptability were found both before and after (median range 7.0–7.0, IQR 2.0–0.0 and 5.0–7.0, IQR 2.0–2.0, respectively) the intervention.
Recio-Rodriguez et al. (34)	To assess the efficacy of the combined use of smartphone and smart band technology for 3-months alongside brief lifestyle counseling, versus counseling alone, in increasing physical activity. As secondary objectives, the effects of the intervention on dietary habits, body composition, quality of life, level of functionality and cognitive performance were assessed.	Both groups received brief nutritional advice (aimed at good adherence to the Mediterranean Diet) and physical activity advice. Intervention group participants were instructed to use a smartphone application for a period of 3-months. This application integrates information on physical activity received from a fitness bracelet and self-reported information on the patient’s daily nutritional composition. At the end of the day, the application makes recommendations and a personalized plan to improve eating habits and physical activity over the following days	Adherence to the Mediterranean diet: (the Mediterranean Diet Adherence Questionnaire): no difference Caloric intake: no difference Body fat percentage: no difference Quality of life (WHOQOL-AGE): no difference	Adherence to the intervention: the mean number of days the application was used by the intervention group was 70.7 ± 21.3, an adherence to the intervention of 78.5% ± 23.7%.
Van Asbroeck et al. (26)	To investigate whether online education about dementia risk reduction may be a low-level means to increase knowledge and support self-management of modifiable dementia risk factors.	The e-learning consists of seven parts that are delivered via weekly emails. The seven parts cover the following topics: (1) how the brain works and cognitive changes during aging, (2) cognitive and social activity, (3) healthy diet and alcohol consumption, (4) physical activity, (5) mental wellbeing, including sleep, (6) cardiovascular health, and (7) lifestyle coach-guided advice on how to make sustainable lifestyle changes. The weekly email guides users first to a short knowledge quiz, and thereafter to a webpage containing further information, including a video, and practical tips. The email further contains a selection of three weekly challenges (ranging in difficulty from light to hard) as an immediate call to action.	Motivation for health behaviors: motivation for a healthy diet or reducing alcohol consumption did not change over time. Adherence to the Mediterranean diet: increased over time (b0 = 0.27, *p* = 0.002 and b3 months = 0.32, *p* = 0.001). Alcohol consumption: no overall effect of time on alcohol consumption was observed, yet at three-month follow-up, alcohol consumption was significantly decreased in women compared to men (b = −1.21, *p* = 0.008).	User experience: the e-learning’s overall quality scored 8.2 out of 10 (SD = 1.8) for ease of use, 7.7 out of 10 (SD = 1.8) for attractiveness and enjoyability, and 7.6 out of 10 (SD = 1.8) for the overall perceived educational and motivating quality. Participants rated the e-learning a 7.0 out of 10 (SD = 2.3) for meeting their expectations. Women consistently rated the e-learning higher on all four of these dimensions (all *p* < 0.050). Individuals between 61 and 70 years old rated e-learning higher for meeting their expectations (7.3 versus 6.6, *p* = 0.034), attractiveness and enjoyability (8.0 versus 7.4, *p* = 0.028), and educational and motivating quality (7.9 versus 7.2, *p* = 0.010), compared to older individuals (>70 years old). User experiences were positive with weekly themes receiving average ratings between 7.9 and 8.1 out of 10. Engagement: overall was high but appeared to decline over time.
van Doorn-van Atten et al. (27)	To evaluate the effects of an intervention including nutritional telemonitoring, nutrition education, and follow-up by a nurse on nutritional status, diet quality, appetite, physical functioning and quality of life of Dutch community-dwelling elderly.	Participants’ TVs had an extra channel with menus for a schedule, messages, results, and health advice. Participants daily received 1–3 TV messages, some tailored, regarding diet and exercise. Non-tailored messages utilize behavioral strategies, including belief selection and awareness. Personalized digital messages conveyed the Dutch Healthy Diet Food Frequency Questionnaire results and guidance for enhancing diet and activity. Telemonitoring data was transmitted via a set-top box to a healthcare website, reviewed weekly by three nurses. If the participant risked undernutrition, the nurse advised on boosting protein and energy intake, providing an advice brochure.	Nutritional status (Mini Nutritional Assessment Short-Form, MNA-SF): improved for participants with poor nutritional status compared to the controls (β = 1.77; 95 % CI 0.60, 2.94). No difference for participants with a normal nutritional status. Compliance with Dutch guidelines improved for the intake of vegetables (β = 1.27; 95 % CI 0.49, 2.05), fruit (β = 1.24; 95 % CI 0.60, 1.88), dietary fiber (β = 1.13; 95 % CI 0.70, 1.57), and protein (β = 1.20; 95 % CI 0.15, 2.24). Slightly decreased for the intake of Na, whereas participants in the control group increased their compliance (β = −0.97; 95% CI −1.77, −0.17). No effect for the intake of fish, saturated-fatty acids, trans-fatty acids, alcohol and vitamin D, total score for diet quality, body weight, appetite, physical functioning and quality of life.	–
van Doorn-van Atten et al. (23)	To evaluate the feasibility of telemonitoring intervention to improve the nutritional status of community-dwelling older adults.	Participants’ TVs had an extra channel with menus for a schedule, messages, results, and health advice. Participants daily received 1–3 TV messages, some tailored, regarding diet and exercise. Non-tailored messages utilize behavioral strategies, including belief selection and awareness. Personalized digital messages conveyed the Dutch Healthy Diet Food Frequency Questionnaire results and guidance for enhancing diet and activity. Telemonitoring data was transmitted via a set-top box to a healthcare website, reviewed weekly by three nurses. If the participant risked undernutrition, the nurse advised on boosting protein and energy intake, providing an advice brochure.	Scores for compliance with dietary guidelines improved for fish (M1–M0 = 2.8 [1.3, 4.3]), dietary fiber (M1−M0 = 1.2 [0.01, 2.4]), protein (M1−M0 = 5.2 [2.6, 7.7]), and vitamin D (M1−M0 = 0.6 [0.1, 1.1]). Score for compliance with the guideline for saturated fatty acids decreased (M1−M0 = −3.3 [−5.9, −0.6]). No significant changes in most of the behavioral determinants of healthy eating (self-developed questionnaire), diet quality (DHD-FFQ), body weight, appetite (SNAQ), nutritional status (Mini Nutritional Assessment, MNA) and quality of life (SF-36) scores.	Acceptability: half of the participants agreed that they were satisfied with the project and one-third was neutral about this statement. Some participants perceived the project as a heavy burden on their daily lives, were puzzled by the message that they risked undernutrition, and found the dietary advice not personal enough or sounding “unfriendly” (themes experience with dietary advice and risk of undernutrition). Usability: the project was rated lower in terms of attractiveness, clarity, ease of navigation, and ease of obtaining overview. Participants experienced stress and frustration when the channel did not work properly, and some participants became insecure about their own capabilities.

A narrative synthesis was conducted because of substantial differences in the studies’ measurement methods. A meta-analysis could not be performed due to the disparate measurement units among the various studies; consequently.

### Quality assessment of the included studies

A quality assessment was performed for quantitative studies; in particular, the Risk of Bias 2 (RoB 2) tool (20) and the Risk of Bias Assessment Tool for Non-randomized Studies (RoBANS 2) tool (21) were used for a critical assessment of the methodological quality of the included randomized studies and non-randomized studies of intervention, respectively (Supplementary Appendix B).

## Results

### Identified studies

**FIGURE 1:**
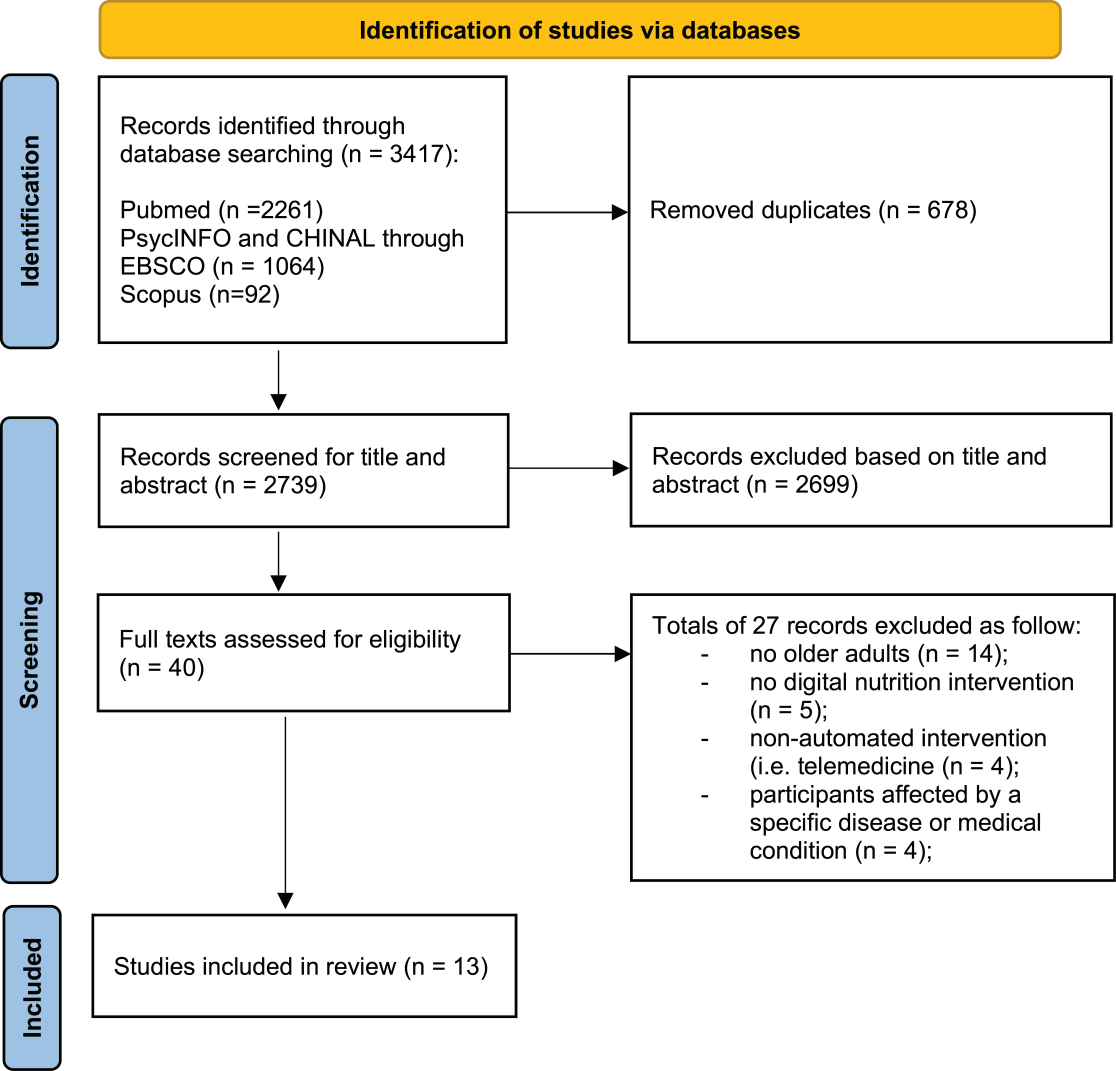
The Preferred Reporting Items for Systematic Reviews and Meta-Analyses (PRISMA) diagram of the article search (18).

The initial search yielded a total of 3,417 citations from the electronic databases. After the removal of duplicates, 2,739 abstracts were screened for eligibility and, among them, 40 papers were assessed in full text. 27 studies were excluded for the following reasons: 14 studies were not conducted on older adults; five studies were not digital nutrition interventions; four studies reported telemedicine interventions (non-automated); and four studies included participants affected by specific medical conditions. Ultimately, 13 studies met the eligibility criteria and were included in the systematic review. Figure 1 details the search process.

### Characteristics of the included studies

General characteristics of the included studies are reported in Table 1. The articles were published from 2016 to 2025; the temporal distribution of the publications does not show a clear increasing or decreasing trend but is evenly distributed with a peak of three publications in 2019 (22–24).

Almost all of the included studies (12 out of 13) were based in Europe; in particular, five studies were conducted in Netherlands (22, 23, 25–27), four in Norway (28–31), one in both the Netherlands and in Portugal (32), one in Portugal (33), and one in Spain (34); the only study outside Europe was conducted in Taiwan (24).

The sample sizes ranged from 4 (26) to 477 (26) participants, with a higher representation of women in all but one study (28). The mean age of the samples ranged from 63 (26) to 81 years (23, 30).

The duration of the interventions ranged from 4 weeks (29) to 14 months (32); in particular, six studies lasted between 1 and 2 months (22, 24, 26, 29–31), six studies lasted between 2 and 9 months (23, 25, 27, 28, 33, 34), and one study lasted 14 months (32).

Among the nine interventions reporting quantitative analyses, two were randomized controlled trials (22, 34) and seven had a before-and-after design (23–27, 32, 33); four of them also conducted a qualitative evaluation of the intervention (23–25, 32). Four studies exclusively performed qualitative analyses (28–31).

Three interventions were delivered in a fully automated mode (26, 32, 33), while the other 10 studies (22–25, 27–31, 34) were blended and involved dedicated staff, particularly during initial meetings or follow-up.

Although each intervention possessed distinct characteristics, which are detailed in Table 2, they can be categorized based on their mode of delivery as follows: nine studies (20, 24–31) required participants to utilize applications offering meal recommendations, often personalized, along with nutritional guidance; three of these applications were television-based (20, 24, 30); two studies delivered an e-learning course on nutrition (22, 23); one intervention provided online tailored recommendations (19); and a single study supplied participants with a tablet featuring more than 50 nutrition-related films and applications (21).

### Quantitative findings

Quantitative outcomes assessed by two or more studies have been synthesized and reported in Table 3. Fourteen different outcomes were identified: nutritional status, self-perceived health/physical function, quality of life, appetite, weight/BMI, body fat percentage, adherence to the Mediterranean diet, calorie intake, protein intake, dietary fiber, fish intake, saturated fatty acids intake, vitamin D intake, and alcohol consumption.

**TABLE 3:** Quantitative outcomes of the included studies.

Outcome variables	Studies ID (main author, year) and results			
Nutritional status	Van Doorn-Van Atten et al. (27): improved for individuals with poor nutritional status at baseline (β = 1.77; 95 % CI 0.60, 2.94)	Van Doorn-Van Atten et al. (23): no difference	–	–
Self-perceived health/physical function	Doets et al. (22): no difference	Gomes et al. (33): improved (mean −0.22, SD 0.38; *P* = 0.01)	van Doorn-van Atten et al. (27): no difference	–
Quality of life	Gomes et al. (33): no difference	Recio-Rodriguez et al. (34): no difference	van Doorn-van Atten et al. (27):no difference	van Doorn-van Atten et al. (23): no difference
Appetite	van Doorn-van Atten et al. (27): no difference	van Doorn-van Atten et al. (23): no difference	–	–
Weight/BMI	Bohn et al. (32): no difference	van Doorn-Van Atten et al. (27): no difference	van Doorn-van Atten et al. (23): no difference	–
Body fat percentage	Bohn et al. (32): no difference	Recio-Rodriguez et al. (34): no difference	–	–
Adherence to the Mediterranean diet	Gomes et al. (33): no difference	Recio-Rodriguez et al. (34): no difference	van Asbroeck et al. (26): increased over time (b0 = 0.27, *p* = 0.002 and b3 months = 0.32, *p* = 0.001).	–
Calorie intake	Doets et al. (22): no difference (no quantitative data available)	Recio-Rodriguez et al. (34): no difference	–	–
Protein intake	Doets et al. (22): decreased (no quantitative data available)	Dorhout et al. (25): no difference	van Doorn-van Atten et al. (27): increased β = 1.20; 95 % CI 0.15, 2.24	van Doorn-van Atten et al. (23): increased M1−M0 = 5.2 [2.6, 7.7]
Dietary fiber	van Doorn-van Atten et al. (27): improved (β = 1.13; 95 % CI 0.70, 1.57)	van Doorn-van Atten et al. (23): improved M1−M0 = 1.2 [0.01, 2.4]	–	–
Fish intake	van Doorn-van Atten et al. (27): no difference	van Doorn-van Atten et al. (23): increased M1−M0 = 2.8 [1.3, 4.3]	–	–
Saturated fatty acids intake	van Doorn-Van Atten et al. (27): no difference	van Doorn-van Atten et al. (23): decreased M1−M0 = −3.3 [−5.9, −0.6]	–	–
Vitamin D intake	Doets et al. (22): no difference (no quantitative data available)	van Doorn-van Atten et al. (27): no difference	Van Doorn-Van Atten et al. (23): increased M1−M0 = 0.6 [0.1, 1.1]	–
Alcohol consumption	van Asbroeck et al. (26): decreased in women (b = −1.21, *p* = 0.008), no difference in men	van Doorn-van Atten et al. (27): no difference	–	–

The included interventions did not prove effectiveness for five of these outcomes; in particular, no difference was found for quality of life [*n* = 4 studies (23, 27, 33, 34)], appetite [*n* = 2 studies (23, 27)], weight/BMI [*n* = 3 studies (23, 27, 32)], body fat percentage [*n* = 2 studies (32, 34)], and calorie intake [*n* = 2 studies (22, 34)].

The results concerning the other outcomes were mixed. Among the two studies evaluating changes in nutritional status, one (27) reported that individuals with poor nutritional status at baseline improved their nutritional status at the end of the intervention, and the other (23) found no difference. Self-perceived health/physical function improved in one study (33) but not in two other interventions (22, 27). Adherence to the Mediterranean Diet was assessed in three studies; two did not show significant effects of the intervention (33, 34), while the third showed an increase over time (26). Four studies evaluated the intervention’s effectiveness in increasing protein intake: two reported increased protein intake (20, 24), one found no difference (25), and one found decreased protein intake (22). Two studies conducted by the same authors addressed the intervention’s effectiveness in improving adherence to Duch nutritional guidelines (23, 27); the results showed improvement post intervention for dietary fiber, and mixed results for fish intake [one study (23) showed improved adherence to the guidelines and the other found no effectiveness (27)], and saturated fatty acid intake [decreased in one study (23) and no difference in another (27)]. Vitamin D intake was assessed by three studies; two of them (22, 27) found no effectiveness, and one (23) reported improvement post-intervention. Finally, one study evaluating the effects of the intervention regarding alcohol consumption showed that it decreased in women (26) but not in men, and another study found no effectiveness in decreasing alcohol consumption (27).

### Qualitative findings

The qualitative analysis of the included studies identified seven recurring themes, as described in Table 4: ease of use, dropouts, education and awareness, inspiration and motivation, acceptability, burden, and technical issues.

**TABLE 4:** Qualitative outcomes of the included studies.

Identified themes	Description
Ease of use	Some apps were considered complex to use (23), especially for the older ones (28), or not user-friendly (22), while others were appreciated for their ease of use (26, 29, 31).
Acceptability	The acceptability of the interventions was considered good for some of the studies (23, 33), while others were less accepted (32).
Dropouts	The dropout rates were generally quite high and increased especially toward the end of the interventions (25, 26, 30, 32).
Education and awareness	Many participants stated that the educational content of the interventions was significant (22, 24, 33) and that recording their meals made them more aware of their food intake (22, 29–31).
Inspiration and motivation	The meal suggestions were considered inspirational and motivated people to adopt a healthy diet (26, 29, 30, 33).
Burden	Some interventions were considered to have a heavy burden on participants’ daily lives (23), and recording meals on apps can appear restrictive (31).
Technical issues	Participants often needed technical support (30, 31) or experienced frustration from technical problems (23).

Ease of use was generally considered a very important aspect of the interventions; three apps were considered easy to use (23, 26, 28), while three others were evaluated as not user-friendly (19) or complex to use (20), particularly by older participants (28). Similarly, the interventions’ acceptability ratings were high in two studies (23, 33), though it was lower in another (32). Participants’ dropout rates were generally elevated and showed a marked increase near the conclusion of the interventions (25, 26, 30, 32). Many participants rated the educational content of the interventions positively (22, 24, 33) and reported that recording their meals in the apps made them more aware of their actual food consumption (22, 29–31). In four studies, the opportunity to consult the recommended meals was found to be inspiring, prompting individuals to embrace a healthy eating regimen (26, 29, 30, 33). On the other hand, some participants felt that the interventions placed a heavy burden on their daily lives (23), and recording meals on apps was perceived as restrictive (31). Finally, participants often needed technical support (30, 31) or experienced frustration from technical problems (23).

### Methodological quality of the included studies

The quality assessment of the included studies that performed a quantitative analysis is reported in Supplementary Appendix B. Both randomized controlled trials (22, 34) were judged to have a low risk of bias across all RoB 2 domains (16), indicating strong methodological rigor. In contrast, the before-and-after studies assessed with the RoBANS 2 tool (17) generally showed a higher risk of bias, particularly in the domains of comparability of target groups (23, 26, 27, 32), measurement of the intervention (32), outcome assessment (25, 32), and incomplete outcome data (23, 26, 27). Specifically, five studies presented high or unclear risk in multiple domains (23, 24, 26, 27, 32). These methodological limitations may have introduced threats to internal validity and may have downgraded the overall strength and consistency of the evidence.

## Discussion

This scoping review aimed to map and characterize the existing evidence on digital nutrition interventions aimed at improving older adults’ eating behaviors. Overall, the findings highlighted substantial heterogeneity in study design, intervention features and outcome assessments. The limited number of included studies (*n* = 13) suggests that research specifically focusing on digital nutrition interventions for healthy older adults remains scarce. This may reflect both the novelty of digital health approaches in this population and the strict eligibility criteria adopted, particularly the exclusion of studies conducted on seniors affected by a specific disease or medical condition. As a result, the available evidence appears fragmented and still underdeveloped.

From a geographical perspective, the strong concentration of studies conducted in Europe, particularly in the Netherlands and Norway, indicates a potential imbalance in research production. This raises questions about the transferability of the findings to other cultural contexts, especially considering that dietary habits, digital literacy, and access to technology may vary substantially across regions.

The included studies also showed wide variability in sample size, age range, and gender distribution. Most samples were relatively small and predominantly female, which may limit the representativeness of findings.

A key finding of this review is the marked heterogeneity in intervention characteristics. Digital interventions ranged from fully automated applications to blended approaches involving human support, and included different formats such as mobile apps, television-based platforms, and e-learning tools. The predominance of blended interventions suggests that human support may still play a crucial role in facilitating engagement with digital tools in this population.

The study design, the duration of the interventions and outcome measures also varied considerably, which further limits comparability across studies and the reliability of conclusions.

Concerning the available evidence of digital nutrition interventions’ effectiveness, the included studies found no significant differences in weight/BMI (23, 27, 32), body fat percentage (32, 34), or calorie intake (19, 31) after the interventions or between intervention and control groups. This result is not surprising, as the studies included did not specifically select participants who were malnourished or overweight and therefore needed to change their energy intake and weight. On the contrary, it would have been desirable to observe improvements in nutritional status and adherence to nutritional guidelines, but this did not occur consistently (23, 26, 27, 33, 34). The included studies yielded mixed results regarding adherence to the Mediterranean diet (26, 33, 34) and healthy eating guidelines (23, 27), with fewer than half of the studies reporting positive outcomes of the interventions, and the others denying that the interventions had any significant effect. The only outcome variable for which both studies that analyzed it reported a positive effect of the intervention was dietary fiber intake (23, 27). This result is in line with previous research analyzed in a systematic review and meta-analysis, which revealed that computer-tailored communication was effective in increasing fruit and vegetable intake among adults aged forty and over, showing a significant effect size (15).

There may be several reasons why digital nutrition interventions have not proven sufficiently effective in improving the other analyzed outcomes. Firstly, nutrition is an area in which it is difficult to intervene effectively to promote behavioral change, even when using more traditional face-to-face methods and targeting a younger population (35, 36). In fact, modifying eating habits is difficult because many interacting factors influence food choices, including general, environmental, and personal factors that together create a complex system (37). Research has reported that nutritional interventions often fail and do not lead to lasting behavioral changes (38, 39). In fact, given the inherent obstacles in ceasing maladaptive health behaviors and commencing adaptive ones, the intention to change dietary habits on its own does not consistently correlate with the eventual manifestation of behavioral change (40). The likelihood of an individual successfully translating intentions into actions is determined by a combination of internal factors, such as beliefs, skills, and knowledge, and external factors, including available food, monetary resources, and social support (41). In this context, the intention-behavior gap describes the difference between individuals’ stated intentions and their actual actions (35). This gap was clearly highlighted in the study conducted by Doets et al. (22), which showed that although many participants expressed their intention to optimize their calorie or protein intake, insufficient corresponding nutritional changes were observed during the study period.

Moreover, older adults seem to have lower acceptance of digital nutrition interventions than younger populations (42). In this regard, qualitative analysis of the included studies revealed that the dropout rate was high and tended to increase over time (25, 26, 30, 32), thereby contributing to reduced intervention effectiveness. In addition, some studies reported that participants experienced technical problems that forced them to seek support and caused frustration (23, 30, 31). These outcomes are supported by other evidence from the literature, which reports that people aged 50 years and above tend to use nutrition and fitness apps at lower rates and experience more negative attitudes towards these apps, suggesting the existence of a digital divide (42).

Other negative aspects highlighted by the qualitative studies included in the systematic review were the excessive burden the interventions placed on participants’ daily lives and the restrictions some individuals experienced due to the need to record their food intake in apps (23, 31). In fact, food tracking, which is a feature often found in nutrition apps, requires detailed entries that users (of all ages) often perceive as too complex or time-consuming (43). In this regard, nutritional research highlights how food monitoring is associated with a series of potential negative cognitive and emotional consequences, such as obsession with food or calorie counting, excessive desire to control one’s physical state and behaviors, and negative emotional reactions such as guilt and anxiety when a set goal is not achieved (43).

In addition to the negative aspects already listed, participants also highlighted some positive aspects resulting from their participation in the interventions. In particular, older adults appreciated the increased nutritional knowledge (22, 24, 33), the greater awareness of their dietary intake (22, 29–31), and the inspirational and motivational content of the interventions (26, 29, 30, 33). Contrary to behavioral change outcomes, increased nutrition knowledge is usually the most successful outcome reported in nutritional interventions targeting older adults, as previously reported in a review (44). Studies have demonstrated that enhanced awareness of individual health behavior may encourage a shift in behavior, such as adhering to a healthier eating plan (45). For behavior change interventions to succeed, strong motivation to engage in goal-directed actions is critical; however, the capacity to convert this motivation into action is equally essential. The concept in question is likewise described as self-regulation (46, 47). Prior investigations have demonstrated the critical role of self-regulation in both initiating health-promoting behaviors, such as consuming fruits and vegetables, and inhibiting health-compromising behaviors, such as consuming saturated fat-rich products. Consequently, further research is recommended to explore the personalization of dietary advice based on self-regulation determinants, given its significant influence on long-term adherence.

Finally, the findings of this scoping review point to several gaps that warrant further investigation, including the absence of studies from low- and middle-income countries, limited standardization of outcome measures across studies with many relying on self-report data, and the heterogeneity in study design with a paucity of randomized controlled trials which may induce bias and limit the reliability. An additional critical aspect concerns dropout rates and usability challenges, which emerged across several included studies but were not consistently analyzed in relation to intervention outcomes. Future research should implement strategies for increasing adherence to the programs, such as tailoring the interventions to the needs of participants, simplifying interfaces, offering technical support, and integrating motivational components, such as feedback, reminders, and opportunities for social interaction (48–50). Also, actively involving older adults in the development and refinement of interventions and reaching participants during follow-up to investigate the reasons for dropouts may help to develop more user-centered programs (12, 51).

### Limitations

This systematic review has some limitations. First, although the search strategy was developed in accordance with PRISMA guidelines, the involvement of an expert librarian may have further enhanced the comprehensiveness and precision of the database queries. Second, the absence of a third independent reviewer may have limited the ability to further reduce subjectivity and strengthen inter-rater reliability. Although these procedures are commonly adopted in evidence synthesis, their limitations should be considered when interpreting the findings. Third, while the narrow selection criteria helped facilitate comparison and increased confidence in the results, they also reduced the number of eligible studies, possibly excluding relevant ones. Additionally, the small number of studies per outcome requires careful interpretation of the findings. Fourth, although all included studies used digital nutrition interventions and involved older adults, they varied considerably in participant characteristics (such as cultural background, age, and health status), intervention design (including mode of delivery, content, and duration), and outcome measures. This high heterogeneity limited the review’s ability to provide more detailed analysis and prevented a quantitative evaluation of the interventions’ effectiveness. Lastly, the studies have inherent limitations, as they depend on self-reported dietary data, which is common in nutritional research. Participants might misreport their intake due to memory errors, social desirability bias, or inaccurate portion size estimation, potentially leading to inaccurate results (46).

### Conclusion

Digital nutrition interventions have demonstrated effectiveness in enhancing dietary fiber intake among the elderly population. However, no significant impact was observed concerning quality of life, appetite, or caloric intake. The findings regarding other assessed outcomes were mixed. Notably, there was no definitive evidence to support the efficacy of digital interventions in improving nutritional status, self-perceived health, adherence to the Mediterranean diet or dietary guidelines, and protein consumption.

Dropout rates were generally elevated, particularly towards the conclusion of the interventions. Qualitative analyses identified several negative factors, including the complexity of app usage, low acceptability, restrictions and the substantial burden imposed on daily routines by the interventions, as well as frustration stemming from technical issues. Conversely, participants valued increased knowledge, heightened awareness of nutrition, and the motivational and inspirational content provided.
