# Supplementary material for: Digital health interventions for improving nutritional behaviors in older adults: a scoping review
Source: Front Nutr. 2026 May 4;13:1743189. doi: 10.3389/fnut.2026.1743189 (PMC13180575; doi:10.3389/fnut.2026.1743189)
Supplement: Supplementary file 1 [file Supplementary_file_1.docx]

**Supplementary Appendix A.** Research search strings

**PubMed search string:**

(("old people"[Title/Abstract] OR "old adult*"[Title/Abstract] OR "old age"[Title/Abstract] OR "older people"[Title/Abstract] OR "older adult*"[Title/Abstract] OR "older age"[Title/Abstract] OR "geriatric*"[Title/Abstract] OR "elder*"[Title/Abstract] OR "senior*"[Title/Abstract] OR "older person*"[Title/Abstract] OR "old person*"[Title/Abstract] OR "aging adult*"[Title/Abstract] OR "aging person*"[Title/Abstract] OR "Aged"[MeSH Terms]) AND ("telemedicine"[MeSH Terms] OR "telemedicine"[Title/Abstract] OR "Mobile Health"[Title/Abstract] OR "Telehealth"[Title/Abstract] OR "mHealth"[Title/Abstract] OR "eHealth"[Title/Abstract] OR "m-health"[Title/Abstract] OR "e-health"[Title/Abstract] OR "SMS"[Title/Abstract] OR "MMS"[Title/Abstract] OR "digital"[Title/Abstract] OR "smartphone"[Title/Abstract] OR "smartphones"[Title/Abstract] OR "Cell Phone"[Title/Abstract] OR "Cellular Phone"[Title/Abstract] OR "Telephone"[Title/Abstract] OR "Mobile Phone"[Title/Abstract] OR "iPhone"[Title/Abstract] OR "Cell Phones"[Title/Abstract] OR "Cellular Phones"[Title/Abstract] OR "Telephones"[Title/Abstract] OR "Mobile Phones"[Title/Abstract] OR "online"[Title/Abstract] OR "internet"[Title/Abstract] OR "web"[Title/Abstract] OR "Message"[Title/Abstract] OR "Messages"[Title/Abstract] OR "Messaging"[Title/Abstract] OR "Text-Messaging"[Title/Abstract] OR "Texting"[Title/Abstract] OR "Email"[Title/Abstract] OR "E-Mail"[Title/Abstract] OR "Electronic Mail"[Title/Abstract] OR "Emails"[Title/Abstract] OR "E-Mails"[Title/Abstract] OR "Mobile Application"[Title/Abstract] OR "Mobile Applications"[Title/Abstract] OR "Mobile Apps"[Title/Abstract] OR "Mobile App"[Title/Abstract] OR "App"[Title/Abstract] OR "Smartphone Apps"[Title/Abstract] OR "Smartphone App"[Title/Abstract] OR "notification"[Title/Abstract] OR "notifications"[Title/Abstract] OR "reminder"[Title/Abstract] OR "alert"[Title/Abstract] OR "digital technolog*"[Title/Abstract] OR "information technolog*"[Title/Abstract] OR "information communication technolog*"[Title/Abstract] OR "video call*"[Title/Abstract] OR "videoconference*"[Title/Abstract] OR "video conference*"[Title/Abstract] OR "videophone*"[Title/Abstract]) AND ("Diet"[MeSH Terms] OR "diet*"[Title/Abstract] OR "meal plan*"[Title/Abstract] OR "food education"[Title/Abstract] OR "caloric intake*"[Title/Abstract] OR "protein intake*"[Title/Abstract] OR "nutrient*"[Title/Abstract])) AND ((y_10[Filter]) AND (humans[Filter]) AND (english[Filter]) AND (aged[Filter]))

**PsycINFO and CINAHL search string, EBSCO database:**

AB ("old people" OR "old adult*" OR "old age" OR "older people" OR "older adult*" OR "older age" OR "geriatric*" OR "elder*" OR "senior*" OR older person* OR old person* OR aging adult* OR aging person* OR "Aged") AND AB ( telemedicine OR telemedicine OR "Mobile Health" OR "Telehealth" OR "mHealth" OR "eHealth" OR "m-health" OR "e-health" OR "SMS" OR "MMS" OR "digital" OR "smartphone" OR "smartphones" OR "Cell Phone" OR "Cellular Phone" OR "Telephone" OR "Mobile Phone" OR "iPhone" OR "Cell Phones" OR "Cellular Phones" OR "Telephones" OR "Mobile Phones" OR “online” OR “internet” OR “web” OR "Message" OR "Messages" OR "Messaging" OR "Text-Messaging" OR "Texting" OR "Email" OR "E-Mail" OR “Electronic Mail” OR "Emails" OR "E-Mails" OR "Mobile Application" OR "Mobile Applications" OR "Mobile Apps" OR "Mobile App" OR "App" OR "Smartphone Apps" OR "Smartphone App" OR "notification" OR "notifications" OR "reminder" OR "alert" OR “digital technolog*” OR “information technolog* OR “information communication technolog*” OR “video call*” OR “video conference* OR “videoconference” OR “videophone*”) AND AB (“diet*” OR “meal plan*” OR “food education” OR “caloric intake*” OR “protein intake*” OR “nutrient*”). Limiters: English; 2015 - 2025; Aged (65 yrs & older); Human

**Scopus search string:**

( TITLE-ABS ( old AND people ) OR TITLE-ABS ( old AND adult* ) OR TITLE-ABS ( old AND age ) OR TITLE-ABS ( older AND people ) OR TITLE-ABS ( older AND adult* ) OR TITLE-ABS ( older AND age ) OR TITLE-ABS ( geriatric* ) OR TITLE-ABS ( elder* ) OR TITLE-ABS ( senior* ) OR TITLE-ABS ( older AND person* ) OR TITLE-ABS ( old AND person* ) OR TITLE-ABS ( aging AND adult* ) OR TITLE-ABS ( aging AND person* ) OR TITLE-ABS ( ageing AND adult* ) OR TITLE-ABS ( ageing AND person* ) OR TITLE-ABS ( aged ) ) AND ( TITLE-ABS ( telemedicine ) OR TITLE-ABS ( mobile AND health ) OR TITLE-ABS ( tele AND health ) OR TITLE-ABS ( e-health ) OR TITLE-ABS ( m-health ) OR TITLE-ABS ( SMS ) OR TITLE-ABS ( MMS ) TITLE-ABS ( digital ) OR TITLE-ABS ( smartphone ) TITLE-ABS ( smartphones ) OR TITLE-ABS ( cell-phone ) OR TITLE-ABS ( cellular-phone ) OR TITLE-ABS ( telephone ) OR TITLE-ABS ( mobile-phone ) OR TITLE-ABS ( iPhone ) OR TITLE-ABS ( cell-phones ) OR TITLE-ABS ( cellular-phones ) OR TITLE-ABS ( telephones ) OR TITLE-ABS ( mobilephones ) OR TITLE-ABS ( online ) OR TITLE-ABS ( internet ) OR TITLE-ABS ( web ) OR TITLE-ABS ( message ) OR TITLE-ABS ( messages ) OR TITLE-ABS ( messaging ) OR TITLE-ABS ( text-messaging ) OR TITLE-ABS ( texting ) OR TITLE-ABS ( email ) OR TITLE-ABS ( e-mail ) OR TITLE-ABS ( electornic-mail ) OR TITLE-ABS ( emails ) OR TITLE-ABS ( e-mails ) OR TITLE-ABS ( mobile AND application ) OR TITLE-ABS ( mobile AND applications ) OR TITLE-ABS ( mobile AND apps ) OR TITLE-ABS ( mobile AND app ) OR TITLE-ABS ( app ) OR TITLE-ABS ( smartphone AND apps ) OR TITLE-ABS ( smartphone AND app ) OR TITLE-ABS ( notification ) OR TITLE-ABS ( notifications ) OR TITLE-ABS ( reminder ) OR TITLE-ABS ( alert ) OR TITLE-ABS ( digital AND technolog* ) OR TITLE-ABS ( information AND technolog* ) OR TITLE-ABS ( information AND communication AND technolog* ) OR TITLE-ABS ( video AND call* ) OR TITLE-ABS ( videoconference* ) OR TITLE-ABS ( video AND conference* ) OR TITLE-ABS ( videophone* ) ) AND ( TITLE-ABS ( diet* ) OR TITLE-ABS ( meal AND plan* ) OR TITLE-ABS ( food AND education ) OR TITLE-ABS (caloric AND intake* ) OR TITLE-ABS ( protein AND intake* ) OR TITLE-ABS ( nutrient* ) )

**Supplementary Appendix B.** Quality assessment of the included studies according to the RoB 2 tool (20) and the RoBANS 2 tool (21).

| **Main author, year** | | | **RoB 2 domanis** | | | | | | | | | | | | |
| --- | --- | --- | --- | --- | --- | --- | --- | --- | --- | --- | --- | --- | --- | --- | --- |
|  |  |  | **Risk of bias arising from the randomization process** | | **Risk of bias due to deviations from the intended interventions** | | **Missing outcome data** | | | **Risk of bias in measurement of the outcome** | | **Risk of bias in selection of the reported result** | | **Overall risk of bias** | |
| Doets, 2019 (22) | | | Low risk | | Low risk | | Low risk | | | Low risk | | Low risk | | Low risk | |
| Recio-Rodriguez, 2022 (34) | | | Low risk | | Low risk | | Low risk | | | Low risk | | Low risk | | Low risk | |
|  | **RoBANS 2 domanis** | | | | | | | | | | | | | | |
| **Main author, year** | | **Comparability of the target group** | | **Target group selection** | | **Confounders** | | **Measurement of intervention/ exposure** | **Blinding of assessors** | | **Outcome assessment** | | **Incomplete outcome data** | | **Selective outcome reporting** |
| Bohn, 2024 (32) | | High risk | | Low risk | | Low risk | | High risk | Low risk | | High risk | | Unclear risk | | Unclear risk |
| Chiu, 2019 (24) | | Low risk | | Low risk | | Low risk | | Low risk | Low risk | | Low risk | | Unclear risk | | Unclear risk |
| Dorhout, 2025 (25) | | Low risk | | Low risk | | Low risk | | Low risk | Low risk | | High risk | | Low risk | | Low risk |
| Gomes, 2021 (33) | | Low risk | | Low risk | | Low risk | | Low risk | Low risk | | Low risk | | Low risk | | Low risk |
| van Asbroeck, 2025 (26) | | High risk | | Low risk | | Low risk | | Low risk | Low risk | | Low risk | | High risk | | Low risk |
| van Doorn-van Atten, 2018 (27) | | High risk | | Low risk | | Low risk | | Low risk | Low risk | | Low risk | | High risk | | Low risk |
| van Doorn-van Atten, 2019 (23) | | High risk | | Low risk | | Low risk | | Low risk | Low risk | | Low risk | | High risk | | Low risk |
